# Complicações Cardíacas em Longo Prazo da Síndrome do PRKAG2

**DOI:** 10.36660/abc.20210062

**Published:** 2022-01-01

**Authors:** Luiz Pereira de Magalhães, Eduardo Faria Soares de Magalhães, Jussara de Oliveira Pinheiro, Alex Teixeira Guabiru, Francisco José Farias Borges dos Reis, Roque Aras

**Affiliations:** 1 Hospital Universitário Professor Edgard Santos Salvador BA Brasil Hospital Universitário Professor Edgard Santos, Salvador, BA - Brasil; 2 Universidade Federal da Bahia Faculdade de Medicina da Bahia Salvador BA Brasil Universidade Federal da Bahia - Faculdade de Medicina da Bahia, Salvador, BA - Brasil

**Keywords:** Flutter Atrial, Hipertrofia Ventricular Esquerda, Bloqueio Atrioventricular, Marca-Passo Artificial

## Introdução

A síndrome do PRKAG2 é uma doença genética rara, de herança autossômica dominante, causada por mutações no gene que codifica a subunidade γ_2_ da proteína quinase ativada por AMP. A doença está associada ao acúmulo anormal de glicogênio nos cardiomiócitos, predispondo à hipertrofia ventricular, arritmias e morte súbita.^1,2^ A mutação foi primeiramente descrita por Gollob et al. em 2001, despertando, na comunidade médica, a importância do diagnóstico diferencial com miocardiopatia hipertrófica.^3,4^ Embora a prevalência da síndrome do PRKAG2 não esteja estabelecida, o número de casos pode estar aumentando devido à maior disponibilidade de genotipagem. No entanto, ainda não foram descritos possíveis fatores prognósticos na literatura. Considerando a possível gravidade do quadro clínico e a escassez de dados a respeito da história natural, o objetivo deste estudo foi avaliar a evolução clínica de pacientes portadores da síndrome do PRKAG2, analisando a incidência de complicações cardíacas em longo prazo.

## Métodos

Trata-se de estudo observacional, ambispectivo, envolvendo membros de uma única família portadora de mutação Arg302Gln do gene *PRKAG2*.^5^ Foram coletados dados clínicos, e realizados eletrocardiograma (ECG), ecocardiograma, e estudo eletrofisiológico. Hipertrofia ventricular esquerda foi definida como espessura de septo interventricular ou de parede posterior ≥ 13 mm no ecocardiograma, sem causa aparente. O teste exato de Fisher foi utilizado para comparações entre variáveis categóricas, e o teste t de Student para amostras independentes foi usado para comparações entre médias das variáveis contínuas. Foram calculadas as razões de prevalência entre as variáveis de interesse e os desfechos clínicos. O desfecho primário foi implante de marcapasso (MP); o desfecho combinado foi definido pela ocorrência de implante de marca-passo (MP) ou morte súbita. O método de Kaplan-Meier foi utilizado para estimar a incidência cumulativa do desfecho combinado. Um valor de p < 0,05 foi considerado estatisticamente significativo.

## Resultados

**Figura 1:**
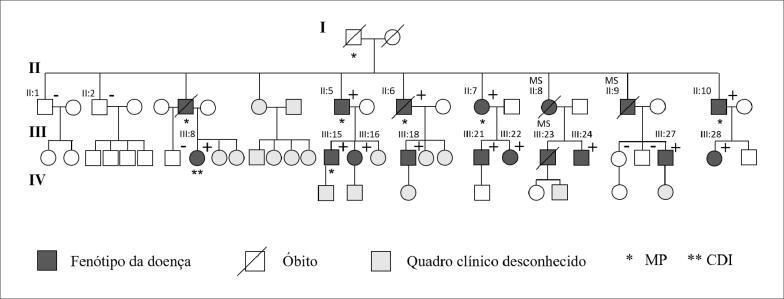
*Heredograma da família. Indivíduos testados quanto à mutação do PRKAG2: (+) portadores; (-) não portadores da mutação. MP: marca-passo; CDI: cardioversor-desfibrilador implantável; MS: morte súbita*.

**Figura 2:**
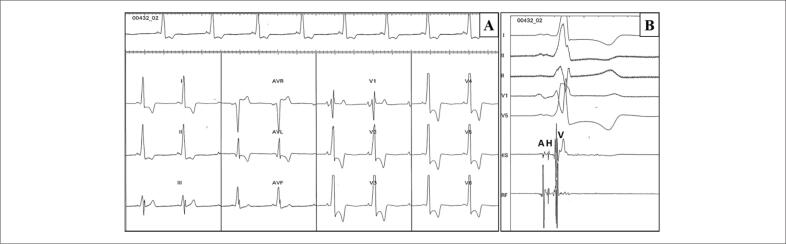
*A) Eletrocardiograma da paciente III:8 mostrando pré-excitação ventricular e morfologia de bloqueio de ramo direito. B) Estudo eletrofisiológico. Intervalo HV= 30 ms. Eletrogramas, A: atrial; H: His; V: ventricular*.

Entre março de 1996 e janeiro de 2020, foram avaliados 16 indivíduos (63% homens, n=10) idade média de 40±11 anos (Figura 1). As características basais dos pacientes estão descritas na Tabela 1. Três indivíduos (18%) apresentaram morte súbita. Durante um tempo médio de acompanhamento de 15,1 ± 2,9 anos, cinco (38%) dos 13 pacientes remanescentes necessitaram de MP devido a bloqueio atrioventricular ou disfunção sinusal, em idade média de 44 ± 6 anos. O fenótipo predominante foi caracterizado por bradicardia sinusal e pré-excitação ventricular, encontrada em todos os pacientes, (Figura 2A); seis (46%) tiveram *flutter* ou fibrilação atrial, e 7 (54%) apresentaram hipertrofia ventricular esquerda ao ecocardiograma. Todos os pacientes apresentaram fração de ejeção preservada. Seis pacientes foram submetidos ao estudo eletrofisiológico, o qual foi consistente com a via fascículo-ventricular (Figura 2B). A medida dos intervalos basais mostrou intervalo HV curto e fixo (mediana=30 ms). Não houve indução de arritmia ventricular em nenhum paciente.

**Tabela 1:** Características clínicas dos pacientes (n=16) de uma única família portadora de mutação Arg302Gln do gene PRKAG2

Paciente	Sexo	Idade	Morte Súbita	Arritmia SV	DCI	HVE
**II:8**	F	38†	+	NA	-	NA
**II:9**	M	40†	+	NA	-	NA
**III:23**	M	28†	+	NA	-	NA
**II:5**	M	56	-	FA, FLA	MP	+
**II:6**	M	60†	-	FA, FLA	MP	+
**II:7**	F	58	-	FLA	MP	+
**II:10**	M	53	-	FLA	MP	+
**III:8**	F	43	-	-	CDI	+
**III:15**	M	31	-	-	MP	-
**III:16**	F	33	-	FA	-	-
**III:18**	M	43	-	FLA	-	-
**III:21**	M	39	-	-	-	+
**III:22**	F	35	-	-	-	-
**III:24**	M	35	-	-	-	-
**III:27**	M	35	-	-	-	+
**III:28**	F	20	-	-	-	-

*Idade em anos; + presente; - ausente;*

† *: óbito, M: masculino; F: feminino; DCI: dispositivo cardíaco implantável; MP: marca-passo; CDI: cardioversor-desfibrilador implantável; HVE: hipertrofia ventricular esquerda; NA: não avaliado; SV: supraventricular; FA: fibrilação atrial; FLA: flutter atrial.*

**Figura 3:**
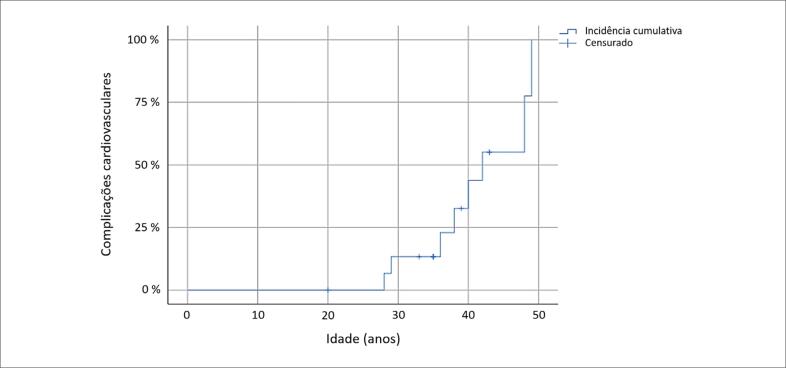
*Método de Kaplan-Meier: incidência cumulativa de complicações cardiovasculares (implante de marca-passo ou morte súbita)*.

A incidência cumulativa do desfecho combinado (implante de MP e morte súbita) está apresentada na Figura 3. Os eventos cardiovasculares ocorreram antes dos 50 anos. A probabilidade de desenvolvimento do desfecho combinado aos 40 anos foi de 44% (IC 95%: 14-84%). Também foram comparadas as características de pacientes submetidos a implante de MP e pacientes sem MP (Tabela 2). *Flutter* atrial foi significativamente mais prevalente nos pacientes que necessitaram de MP (80% *vs* 13%, p=0,032), estando associada a probabilidade 6,4 vezes maior de evolução para esse desfecho.

**Tabela 2:** Análise comparativa de características clínicas entre pacientes com e sem implante de marca-passo

Características	Com marca-passo n= 5	Sem marca-passo n= 8	RR (IC95%)	Valor de p
Idade (anos)	41 ± 8,4	35 ± 7,3	-	0,243
Idade ≥ 40 anos (%)	3 (60)	2 (25)	2,4 (0,6-9,7)	0,293
Sexo masculino (%)	4 (80)	4 (50)	2,5 (0,4-16,5)	0,565
Hipertrofia VE (%)	3 (60)	3 (38)	1,8 (0,4-7,2)	0,592
*Flutter* atrial (%)	4 (80)	1 (13)	6,4 (0,97-42,2)	0,032
Síncope	2 (40)	2 (25)	1,5 (0,4-5,8)	0,999

*VE: ventricular esquerda; RR: risco relativo; IC: intervalo de confiança*.

## Discussão

Analisamos a evolução clínica em longo prazo de portadores de mutação Arg302Gln do gene *PRKAG2* em uma família. Foi observado o caráter precoce de acometimento cardiovascular nesses pacientes, com eventos significativos, como implante de MP ou morte súbita, ocorrendo antes dos 50 anos. Em relação ao eletrocardiograma, todos os pacientes apresentaram pré-excitação ventricular, frequentemente associado a bloqueio de ramo direito e bradicardia sinusal. De acordo com estudos prévios, a prevalência dessas anormalidades é variável e pode aumentar com a idade.^6^ Em nosso estudo, a maioria dos pacientes desenvolveram hipertrofia ventricular ao longo do tempo, porém não encontramos associação desse achado com a indicação de MP. Gollob et al. descreveram complicações cardiovasculares importantes na síndrome do PRKAG2 na ausência de hipertrofia ventricular, sugerindo que essa variável não seja um fator preditor preciso.^7^

Outra característica marcante da síndrome do PRKAG2 é o comprometimento progressivo do sistema de condução cardíaco, com disfunção do nó sinusal e bloqueio atrioventricular.^6^ Em nossa coorte, o implante de MP foi frequente, acometendo 38,5% dos pacientes. Um achado interessante foi a prevalência de *flutter* atrial significativamente maior nesses casos, atingindo 100% dos maiores de 50 anos. Embora essa associação possa ser explicada meramente por coincidência de eventos, parece plausível que o *flutter* atrial desempenhe papel preditor de complicações cardiovasculares.

O mecanismo de morte súbita na doença é controverso; o bloqueio atrioventricular e a fibrilação ventricular são possíveis causas, sendo essa última provocada por fibrilação atrial com condução rápida por via acessória.^8^ A possibilidade de arritmia ventricular parece ser baixa, sem relatos de terapias apropriadas com cardioversor desfibrilador implantável (CDI) na literatura. Em nosso estudo identificamos via acessória fascículo-ventricular, cujo envolvimento em arritmias malignas não foi comprovado.^9^ Além disto, não foi registrado arritmia ventricular durante estudo eletrofisiológico ou monitorização por MP/CDI. Portanto, é provável que o bloqueio atrioventricular total tenha sido a causa de morte nesta família, potencialmente evitável com implante de MP. O desafio que persiste é identificar os pacientes com maior risco de evolução desfavorável e instituir terapia adequada preventiva.

Em conclusão, deve-se suspeitar de síndrome do PRKAG2 em pacientes jovens com pré-excitação ventricular, taquiarritmias atriais e hipertrofia ventricular familiar. A associação significativa entre aparecimento de *flutter* atrial e evolução para implante de marca-passo pode ter um papel no manejo dos portadores da síndrome. Futuros estudos devem esclarecer a importância clínica dessa observação.
