# Supplementary material for: Complicações Cardíacas em Longo Prazo da Síndrome do PRKAG2
Source: Arq Bras Cardiol. 2022 Jan 1;118(1):106–9. [Article in Portuguese] doi: 10.36660/abc.20210062 (PMC8959048; doi:10.36660/abc.20210062)
Supplement: Translation

# Translation: Long-Term Cardiac Complications of PRKAG2 Syndrome

**Authors:** Luiz Pereira de Magalhães, Eduardo Faria Soares de Magalhães, Jussara de Oliveira Pinheiro, Alex Teixeira Guabiru, Francisco José Farias Borges dos Reis, Roque Aras

**Affiliations:** 1 Salvador BA Brazil Hospital Universitário Professor Edgard Santos, Salvador, BA - Brazil; 2 Salvador BA Brazil Universidade Federal da Bahia - Faculdade de Medicina da Bahia, Salvador, BA - Brazil

**Keywords:** Atrial Flutter, Hypertrophy, Left Ventricular, Atrioventricular Block, Pacemaker, Artificial

## Introduction

The PRKAG2 syndrome is a rare autosomal dominant inherited disease caused by mutations in the gene encoding the γ^2^ regulatory subunit of AMP-activated protein kinase.^1,2^ It has been associated with abnormal glycogen accumulation in cardiomyocytes, predisposing to ventricular hypertrophy, arrhythmias, and sudden death. Gollob et al. were the first to describe the mutation in 2001, calling attention to the need for differential diagnosis with hypertrophic cardiomyopathy.^3,4^ Although the prevalence of PRKAG2 syndrome is not established, the number of reported cases may be increasing due to wider availability of genotyping. Despite that, possible prognostic factors have not yet been described in the literature. Given the possible severity of clinical presentations and the scarcity of data regarding the natural history, our objectives were to evaluate the clinical course of patients with PRKAG2 syndrome over time and analyze the incidence of long-term cardiac complications.

## Methods

This observational, ambispective study was conducted with members of a single family with an Arg302Gln mutation in *PRKAG2* gene.^5^ Collection of clinical data, electrocardiography (ECG), echocardiography, and electrophysiological studies were performed. Left ventricular hypertrophy was defined by an interventricular septum or left ventricular posterior wall thickness ≥ 13 mm on echocardiography without any other apparent cause. Differences between means of continuous variables were assessed using the Student's t-test for independent samples, and the Fisher's exact test was used for comparison between categorical variables. Prevalence ratios between the variables of interest and clinical outcomes were calculated. The primary outcome was pacemaker implantation; the composite endpoint was either PM implantation or sudden death. The Kaplan-Meier method was used to estimate the cumulative incidence of the composite outcome. A p-value < 0.05 was considered statistically significant.

## Results

From March 1996 to January 2020, 16 individuals were assessed. Mean age was 40±11 years, and 63% (n=10) were male (Figure 1). Baseline characteristics of the patients are shown in Table 1. Three individuals (18%) experienced sudden death. During a mean follow-up of 15.1 ± 2.9 years, 5 (38%) of the remaining 13 patients needed PM placement due to third-degree atrioventricular block or sinus node dysfunction, at a mean age of 44 ± 6 years. The predominant phenotype was characterized by sinus bradycardia and pre-excitation which was present in all patients (Figure 2A); six patients (46%) had atrial fibrillation or atrial flutter; seven (54%) had left ventricular hypertrophy on echocardiography. All patients had preserved ejection fraction. Six patients underwent electrophysiology study, which was consistent with a fasciculoventricular pathway (Figure 2.B). Measurement of baseline intervals demonstrated a fixed, short HV interval (median = 30 ms). Ventricular arrhythmia was not induced in any patient.

**Figure 1:** *Genogram of the family. Individuals tested for a PRKAG2 mutation: carriers (+) or non-carriers (-). PM: pacemaker; ICD: implantable cardioverter-defibrillator; SD: sudden death*.

**Figure 2:** *A) Electrocardiogram of patient III:8 showing ventricular pre-excitation, and right bundle branch block morphology. B) Electrophysiology study of the same patient. HV interval= 30 ms. Electrograms, A: atrial; H: His bundle; V: ventricular*.

The cumulative incidence of the composite outcome is shown in Figure 3. All cardiovascular events occurred before the age of 50. The probability of developing the combined outcome at 40 years was 44% (95% CI: 14-84%). Characteristics of patients submitted to pacemaker implantation and patients without pacemaker were also compared (Table 2). The prevalence of atrial flutter was significantly higher in patients requiring PM (80% vs. 13%, p=0.032). Atrial flutter was associated with a 6.4-fold likelihood of developing a conduction disorder requiring pacemaker implantation.

**Figure 3:** *Kaplan-Meier curve of the cumulative incidence of cardiovascular events (pacemaker implantation or sudden death)*.

**Table 1:** Clinical features of the study patients (n=16), members of a single family with an Arg302Gln mutation in PRKAG2 gene

Patient	Sex	Age (years)	Age at onset of symptoms (years)	Sudden death	CID	LVH
**II:8**	F	38†	-	+	-	NA
**II:9**	M	40†	-	+	-	NA
**III:23**	M	28†	28	+	-	NA
**II:5**	M	56	30	-	PM	+
**II:6**	M	60†	42	-	PM	+
**II:7**	F	58	40	-	PM	+
**II:10**	M	53	44	-	PM	+
**III:8**	F	43	23	-	ICD	+
**III:15**	M	31	26	-	PM	-
**III:16**	F	33	33	-	-	-
**III:18**	M	43	39	-	-	-
**III:21**	M	39	33	-	-	+
**III:22**	F	35	-	-	-	-
**III:24**	M	35	-	-	-	-
**III:27**	M	35	28	-	-	+
**III:28**	F	20	-	-	-	-

+ *present; -: absent; NA: not available;*

† *deceased; M: male; F: female; CID: cardiac implantable device; PM: pacemaker; ICD: implantable cardioverter-defibrillator; LVH: left ventricular hypertrophy.*

**Table 2:** Comparative analysis of clinical characteristics between patients who received a pacemaker and those who did not

Characteristics	Pacemaker N = 5	No pacemaker N = 8	RR (95%CI)	p-value
Age (years)	41 ± 8.4	35 ± 7.3	-	0.243
Age ≥ 40 years (%)	3 (60)	2 (25)	2.4 (0.6-9.7)	0.293
Male (%)	4 (80)	4 (50)	2.5 (0.4-16.5)	0.565
LV hypertrophy (%)	3 (60)	3 (38)	1.8 (0.4-7.2)	0.592
Atrial flutter (%)	4 (80)	1 (13)	6.4 (0.97-42.2)	0.032
Syncope (%)	2 (40)	2 (25)	1.5 (0.4-5.8)	0.999

*LV: left ventricular; RR: relative risk; CI: confidence interval*.

## Discussion

We analyzed the long-term clinical course of carriers of the Arg302Gln *PRKAG2* mutation in a family. Importantly, we observed early cardiovascular involvement in these patients, with significant events, such as PM implantation or sudden death, occurring before the age of 50 years. Regarding electrocardiographic findings, all patients had a pattern of ventricular pre-excitation, often associated with right bundle branch block and sinus bradycardia. According to previous reports, the prevalence of these abnormalities is variable and may increase with age.^6^ In our study, most patients developed ventricular hypertrophy over time. However, we found no significant association between ventricular hypertrophy and indication of PM. Gollob et al. described significant cardiovascular complications in PRKAG2 syndrome without ventricular hypertrophy, which suggests that this criterion is not an accurate predictor.^7^

One of the hallmarks of PRKAG2 syndrome is the progressive impairment of the electrical conduction system of the heart, with sinus node dysfunction and atrioventricular block.^6^ In our cohort, 38.5% of patients ultimately required a PM. Interestingly, the prevalence of atrial flutter was significantly higher in these cases, reaching 100% in those over age 50. Although this association can be explained merely by coincidence of events, it seems plausible to suppose that atrial flutter plays a role in predicting cardiovascular complications.

The mechanism of sudden death in the disease is controversial, with atrioventricular block and ventricular fibrillation being possible causes, the latter caused by atrial fibrillation with rapid conduction down an accessory pathway.^8^ The possibility of ventricular arrhythmia seems to be low, with no reports of appropriate implantable cardioverter defibrillator therapies in the literature. In our study, we identified a fasciculoventricular pathway, whose involvement in malignant arrhythmias has not been proven.^9^ In addition, no ventricular arrhythmias were recorded during electrophysiological study or monitoring through PM/ICD. Therefore, atrioventricular block was the possible cause of death in this family, potentially preventable with pacemaker implantation. The challenge that remains is to identify patients at greater risk of unfavorable outcomes and institute therapy before an event occurs.

In conclusion, PRKAG2 syndrome should be suspected in young patients with ventricular pre-excitation, atrial tachyarrhythmias, and family ventricular hypertrophy. The significant association between atrial flutter and progression to pacemaker implantation may play a role in the management of patients with the syndrome. Future studies should clarify the clinical relevance of this observation.
